# Population genomic analyses reveal evidence for limited recombination in the superbug *Candida auris* in nature

**DOI:** 10.1016/j.csbj.2022.06.030

**Published:** 2022-06-16

**Authors:** Yue Wang, Jianping Xu

**Affiliations:** Department of Biology, McMaster University, Hamilton, ON L8S 4K1, Canada

**Keywords:** *Candida auris*, Phylogenetic incompatibility, Linkage equilibrium, Cytonuclear disequilibrium, Recombination, Comparative genomics, PI, phylogenetic incompatibility, LE, linkage equilibrium, FGT, four-gamete test, SNP, single nucleotide polymorphism

## Abstract

*Candida auris* is a recently emerged, multidrug-resistant pathogenic yeast capable of causing a diversity of human infections worldwide. Genetic analyses based on whole-genome sequences have clustered strains in this species into five divergent clades, with each clade containing limited genetic variation and one of two mating types, *MTL***a** or *MTL***α**. The patterns of genetic variations suggest simultaneous emergence and clonal expansion of multiple clades of this pathogen across the world. At present, it is unclear whether recombination has played any role during the evolution of *C. auris*. In this study, we analyzed patterns of associations among single nucleotide polymorphisms in both the nuclear and the mitochondrial genomes of 1,285 strains to investigate potential signatures of recombination in natural *C. auris* populations. Overall, we found that polymorphisms in the nuclear and mitochondrial genomes clustered the strains similarly into the five clades, consistent with a lack of evidence for recombination among the clades after their divergence. However, variable percentages of SNP pairs showed evidence of phylogenetic incompatibility and linkage equilibrium among samples in both the nuclear and the mitochondrial genomes, with the percentages higher in the total population than those within individual clades. Our results are consistent with limited but greater frequency of recombination before the divergence of the clades than afterwards. SNPs at loci related to antifungal resistance showed frequencies of recombination similar to or lower than those observed for SNPs in other parts of the genome. Together, though very limited, evidence for the observed recombination for both before and after the divergence of the clades suggests the possibility for continuous genetic exchange in natural populations of this important yeast pathogen.

## Graphical abstract

**Figure:**
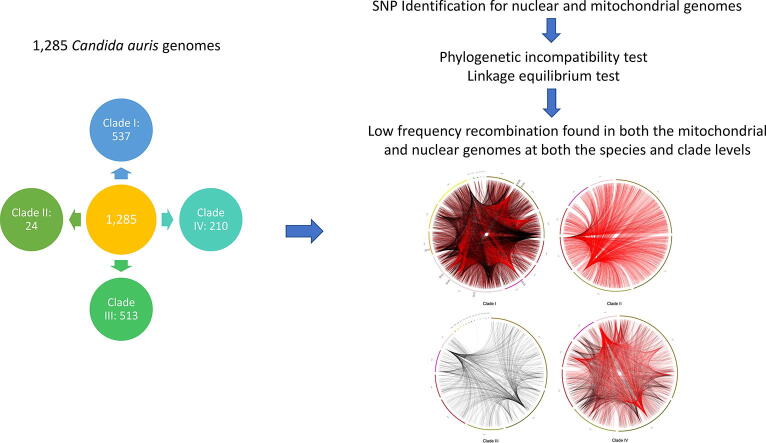

## 1 Introduction

*Candida auris* is a recently emerged yeast pathogen. Since the first report in 2009, *C. auris* has been reported to cause a large number of hospital-related infection outbreaks in many countries, with a high mortality rate [1], [2], [3], [4], [5], [6], [7], [8], [9], [10], [11]. Morphologically and diagnostically, *C. auris* is similar to several other species in the genus *Candida* [12], [13], [14]. However, different from most other pathogenic yeast species, *C. auris* can grow at a high temperature (42 °C) and high salinity conditions, and most strains are resistant to at least one class of antifungal drugs [15], [16]. Other traits of medical importance in *C. auris* include the ability to form biofilm and to persist in hospital environments including medical devices [12]. These unique traits of *C. auris* and its rapid spread have alarmed the World Health Organization and many national public health agencies [17].

Whole-genome sequence analyses have revealed that the global *C. auris* strains belonged to five distinct clades, Clades I to V. While multiple strains have been reported for each of the five clades, only four of the five clades, Clades I to IV, have whole-genome sequences available from multiple strains each. In contrast, the whole-genome sequence of only one Clade V strain is available. The five clades differ from each other by 20,000 to over 200,000 nuclear genome SNPs. In contrast, within each clade, most strains were genetically very similar to each other, with pairwise SNP differences between them ranging from 0 to ∼3,800 SNPs [18], [19], [20], [21]. Such observations suggest that the current global strains were recently derived from a few divergent founder strains, with each underwent rapid clonal expansion and dispersal to result in their current distributions.

So far, no evidence for mating and sexual reproduction have been reported in *C. auris*. Among all strains of *C. auris* analyzed so far, those from Clades I and IV have the *MTL***a** mating type while those in Clades II and III have the alternative *MTL***α** mating type. The lack of alternative mating types within individual clades is consistent with clonal divergence among clades and asexual reproduction within clades in *C. auris.* In addition, within Clade I, all strains sequenced so far contain a deletion of two nucleotides at positions 3,309 and 3,310 at the *STE6* gene. *STE6* is a pheromone transporter, essential for mating in yeasts. The deletion of these two nucleotides makes *STE6* non-functional and consequently the strains in Clade I most likely sterile [22]. Furthermore, several meiosis-specific genes that might have influence on mating and recombination such as *DMC1, RAD55, RAD57,* and *MSH4/5* were not detected in the reference strains of Clades I-IV [20]. Interestingly, *RAD57* was found in the draft genome of a different strain [23], indicating polymorphisms among strains in the distributions of mating and meiosis-specific genes in *C. auris*. Nevertheless, as it has been observed in a related yeast *Candida lusitaniae* [24], [25], the absence of certain mating and meiosis-specific genes should not preclude the possibility that *C. auris* may be capable of mating and sexual reproduction in nature.

Earlier reports of *C. auris* identified geographic clustering among strains of the five clades. Specifically, strains of Clade I were isolated predominantly from South Asia, Clade II predominantly from East Asia, Clade III predominantly from Africa, Clade IV predominantly from the Americas, and several strains of Clade V from Iran in central Asia [9], [21], [26]. However, recent reports showed increasingly broad geographic distributions of clades I, II, III, and IV around the world, with most continents and several countries containing strains belonging to two or more clades. Indeed, strains of different clades have been detected in the same hospitals [27], [28]. Thus, it has been hypothesized that the increasingly mixed distributions of strains with different mating types may create possibilities for mating and recombination among strains of *C. auris* from different clades to generate genetically diverse hybrid offspring [29].

In this study, we analyze the genome-wide allelic associations based on data from over 1,200 strains of *C. auris* to investigate the potential signatures of recombination of this species in nature. The strains were from diverse geographic regions across the world and all known ecological niches such as different body sites in humans, hospital settings, and natural environments. Both the nuclear and mitochondrial genomes were analyzed. Different from the nuclear genome, inheritance of the mitochondrial genome in sexual crosses typically do not follow Mendelian laws, and with both uniparental and biparental inheritance having been observed in ascomycetes [30], [31], [32], [33], [34]. If mating occurred in *C. auris,* the different inheritance mechanisms between the nuclear and mitochondrial genomes will result in cytonuclear genome recombination within and between *C. auris* clades. To investigate the cytonuclear genome recombination, we compared the nuclear and mitochondrial phylogenies. Here, since *C. auris* is a haploid species, associations between alleles at the same SNP site (i.e., the Hardy-Weinberg equilibrium test) cannot be analyzed. Instead, we focus on analyzing associations between alleles at different single nucleotide polymorphism (SNP) sites. Following traditional tests of recombination, pairs of SNP sites that show evidence for phylogenetic incompatibility (PI) are considered as putative evidence for recombination. SNP pairs showing PI are further tested to determine if the relative proportions of the respective genotypes deviate from random association (i.e., in linkage equilibrium, LE). Here, SNP sites from both the nuclear and mitochondrial genomes are identified and analyzed. In addition, we separately analyze SNPs that are shared among clades from those that are found only within individual clades. The details of the population samples, the two tests of recombination, the results and implications of our analyses are described below.

## 2 Materials and methods

### 2.1 Data collection

Whole-genome sequence data for 1,286 *C. auris* strains were downloaded from the National Center for Biotechnology Information (NCBI) Sequence Read Archive. These strains were from five continents, including 673 from the Americas, 141 from Europe, 179 from Africa, 281 from Asia, 10 from Oceania, and two whose location information is unknown. In total, these samples were distributed among 27 countries and were collected from various sources from 1997 up to May 2022. The datasets were from 40 BioProjects that have been linked to 29 studies [5], [15], [18], [20], [21], [35], [36], [37], [38], [39], [40], [41], [42], [43], [44], [45], [27], [46], [47], [48], [49], [50], [51], [52], [53], [54], [55], [56], [57]. Among these 40 BioProjects, eight (including PRJEB21518, PRJNA638416, PRJNA640677, PRJNA657990, PRJNA722434, PRJNA732280, PRJNA772662, and PRJNA796037) currently have no related publications. The details of these strains, including their collection dates, isolation sources, sampling countries, clade affiliations, and reference publications are presented in Table S1.

### 2.2 Genome sequence analysis

To analyze signature of recombination, we first obtained the biallelic SNPs in the genomes of all strains. Here, the completely assembled genome of a commonly used *C. auris* reference strain B8441 (of Clade I) was chosen as the reference to derive the whole-genome SNP dataset for the whole population. The whole-genome SNP dataset was also used to confirm the clade affiliation of each strain. After clade confirmation, strains within each clade were then compared to their corresponding reference strain from the same clade to derive the SNPs for each clade. Specifically, the following four strains were used as references for Clades I-IV respectively, B8441 (GCA_002759435.2; Clade I), B11220 (GCA_003013715.2; Clade II), B11221 (GCF_002775015.1; Clade III), and B11245 (GCA_008275145.1; Clade IV). These four strains were chosen because of their completeness in the genome assemblies and annotations [20]. Because only one Clade V strain has been sequenced at the whole-genome level, the Clade V strain is not included for downstream analyses of recombination and subsequent analyses included the remaining 1,285 strains. SNPs for both the nuclear and mitochondrial genomes were similarly identified using the same four reference strains. However, to date for mitochondrial genome (mitogenome), the NCBI nucleotide database contained only five assembled mitogenomes of *C. auris*, including three circular mitogenomes of Clade I strains (including that of Clade I reference strain B8441 with its mitogenome assembled as MT849287.1), one circular mitogenome of a Clade II strain, and one linear mitogenome of a Clade III strain which likely represents an incomplete mitogenome assembly. Thus, to generate the mitogenome references for Clades II, III and IV, we used raw sequencing reads from strains B11220, B11221, and B11245 and assembled their mitogenomes into circular molecules using NOVOPlasty4.2 [58]. These assembled mitogenome sequences were then used as references to identify mitogenome SNPs among strains within each of the four clades.

For SNP identification, we used the NASP pipeline [59]. Specifically, the following steps were applied to all the strains within each analyzed sample. The adapter sequences and low-quality reads in the raw SRA files were trimmed with Trimmomatic v 0–2.39 [60]. Trimmed reads were then aligned against the selected reference genomes using BWA-MEM v 0.7.17 [61]. SNPs were identified with GATKv2.7 [62]. Finally, for quality insurance, SNP sites were filtered out from specific strains if: (i) they were in repetitive regions of the genomes, (ii) they had read depth lower than 10x, and/or (iii) they had less than 90% of the base calls at the position within the strain.

### 2.3 Phylogenetic analysis

To investigate whether there is evidence for mitochondrial and nuclear genome phylogenetic incongruence at the clade level, we compared the phylogenetic relationships among strains separately derived based on nuclear and mitochondrial genome SNPs. Incongruent relationships among clades between nuclear and mitochondrial genome phylogenies would be consistent with recombination/hybridization among clades. In this analysis, we used strain B8441 as the reference, SNPs of all strains were determined for the nuclear genome. Strains were clustered into representative clades based on nuclear SNPs. Similarly, the mitochondrial SNPs of each strain were determined based on comparison with the mitochondrial genome of strain B8441. Samples with ambiguous calls at over 10% of the SNP sites were removed from the dataset. Then, SNP sites with ambiguous calls of less than 5% of the remaining samples were concatenated. The phylogeny was inferred using FastTree with the GTR+CAT model [63], and visualized with iTOL (https://itol.embl.de/).

### 2.4 PI and LE tests

To investigate evidence for potential recombination between SNPs, we used two approaches that had different null hypotheses [64]. In the first, we determined the prevalence of SNP pairs with PI in the population. In the PI test, the null hypothesis is strict clonality and assumes the absence of parallel mutation. Consequently, in strictly asexual organisms, PI should be absent, and all SNP pairs should be phylogenetically compatible. Specifically, in haploid organisms such as *C. auris*, a pair of SNP sites with two alternative bases each is considered phylogenetically compatible if ≤3 possible genotype combinations are observed in the population. For example, one SNP site has two alternative nucleotides A and G in the population and another SNP site has two alternative nucleotides C and T in the population, if three or fewer of the four possible SNP combinations (AC, AT, GC, and GT) are found in the population, these two SNP sites are considered phylogenetically compatible and be consistent with asexual reproduction in the population. In contrast, if all four possible SNP combinations are found in the population, the two SNPs are considered PI and consistent with recombination between the two SNP sites. [An alternative explanation is parallel mutation at the two SNP sites.] PI is determined using the four-gamete test (FGT). For pairs of SNPs that are PI, we further test whether the observed frequencies of the four genotypes deviate significantly from those expected under random recombination, following the LE test protocol commonly used for haploid organisms (Xu 2006). Different from that of the PI test, the null hypothesis for LE test is random allelic association between SNP sites.

For both tests, we examined the following five datasets. The first dataset contains SNPs in the whole population of 1,285 strains. Here, every SNP site is included if the site has an alternative base in at least one of the 1,285 strains. The SNPs in this dataset are determined based on the alignments of their sequence reads to the genome assembly of strain B8441, the Clade I reference for both the nuclear and mitochondrial genomes. The second dataset consists of four sub-datasets, one for each of four clades and includes nuclear genome SNPs. The third dataset also consists of four sub-datasets, one for each of four clades and includes only the mitogenome SNPs. The fourth dataset again consists of four sub-datasets, one for each of the four clades but included both the nuclear and mitochondrial SNPs where each nuclear SNP was compared with each mitochondrial SNP within each of the four clades in the tests of recombination. The fifth dataset contains only SNPs that are shared among all four clades. In the fifth dataset, each SNP must be polymorphic within each of the four clades.

For each of the above datasets, we first used the FGT to identify pairs of SNP sites that show evidence for PI. The identified PI SNP pairs were then tested for their LE. These tests were performed using in-house scripts written in R3.5 [65]. The *P*-values were adjusted to minimize false discovery rate (FDR) for multiple testing [66]. Circular plot with linked SNPs showing PI was generated using circos-0.69-9 for each of the four individual clades [67].

### 2.5 SNP annotation

As described above, SNP pairs showing evidence of PI and recombination could have been derived due to convergent/parallel mutations among different strains. In general, convergent mutations are more likely to happen to genes under strong selection pressure. To examine this possibility, we annotated the SNPs showing the strongest evidence of recombination to determine whether they are located on genes known to be associated with stress response. We also extracted SNPs from known or putative drug-resistance genes in *C. auris* from the analyzed genomes, using SnpEff5.0 [68]. Both the FGT and LE tests were conducted for the SNPs in these known or putative drug-resistance genes. In addition, the SNPs in these known or putative drug-resistance genes were compared to the SNPs that showed evidence of PI.

## 3 Results

### 3.1 SNP distributions

The whole nuclear genome analysis classified the 1,286 *C. auris* isolates into five clades. This result is consistent with those reported previously (Table S1). Among the 1,286 isolates, 537 isolates belonged to Clade I, 24 belonged to Clade II, 513 belonged to Clade III, 210 belonged to Clade IV, and one belonged to Clade V. The total number of biallelic SNP sites in the nuclear genome for Clades I to V when compared to the Clade I reference strain B8441 were 4,775, 68,248, 48,825, 173,958, and 250,369 respectively. Because there is only one whole-genome sequenced Clade V strain reported so far in the database and this strain is the most divergent among all the strains, we deleted this Clade V strain from subsequent recombination analyses. Interestingly, 13 SNPs were found to be polymorphic within all four clades. Within the four individual clades, the numbers of nuclear SNP sites were 4,775 for Clade I, 3,456 for Clade II, 3,038 for Clade III, and 1,427 for Clade IV (Table 1).

**Table 1:** Numbers of SNP loci for the nuclear and mitochondrial genomes within individual clades and between each of the clades and the Clade I reference strain B8441. For SNPs in the “Clade-specific” column, different clades used different reference genomes for SNP calling, as described in the text.

Reference Feature		Clade-specific	Comparison with Clade I strain B8441
Clade I	Nuclear	4,775	4,775
	Mitochondrial	46	46
Clade II	Nuclear	3,456	68,248
	Mitochondrial	0	12
Clade III	Nuclear	3,038	48,825
	Mitochondrial	19	21
Clade IV	Nuclear	1,427	173,958
	Mitochondrial	0	28
Clade V	Nuclear	N/A	250,369
	Mitochondrial	N/A	60

For the mitochondrial genome, we found a total of 46 SNP sites between B8441 and other strains within Clade I; 12 SNP sites between strain B8441 and those in Clade II; 24 SNP sites between B8441 and those in Clade III; 26 SNP sites between B8441 and those in Clade IV; and 60 SNP sites between B8441 and strain NG-19339 IFRC2087 in Clade V. Different from the nuclear SNPs, none of the mitochondrial SNPs were found to be polymorphic within all four clades. Within individual clades, 46 SNP sites were found in Clade I, 19 SNP sites were detected in Clade III, and no SNP site was found within either Clade II or Clade IV (Table 1).

The mean SNP differences between pairs of strains within each clade for both the mitochondrial and nuclear genomes are summarized in Table 2. To standardize the comparisons between the nuclear and mitochondrial genomes, the mean pairwise SNP frequencies were divided by the reference nuclear and mitochondrial genome sizes (kb) respectively. The mean nuclear SNP differences between pairs of strains within Clades I – IV are 2.45%, 7.71%, 2.36%, and 1.43% respectively. Interestingly, the mitogenomes showed very different patterns from those of the nuclear genomes. In Clades I and III, there was a higher SNP frequency in the mitogenome than in the nuclear genome. However, in Clades II and IV, despite their comparable or higher nuclear SNP frequencies than Clades I and III, no SNP was found in the mitogenomes of either Clade II or Clade IV (Table 2).

**Table 2:** Nuclear and mitochondrial average SNP difference rates within each of Clades I–IV.

	Clade I	Clade II	Clade III	Clade IV
Reference genome size (nuclear; mitochondrial, ×10^6 bp)	12.37; 0.028212	12.25; 0.027071	12.74; 0.028214	12.43; 0.028239
Sample size	537	24	514	210
Nuclear average SNP difference rates	2.45%	7.71%	2.36%	1.43%
Mitochondrial average SNP difference rates	15.96%	0	21.24%	0

### 3.2 Phylogenetic tree

**Fig. 1:**
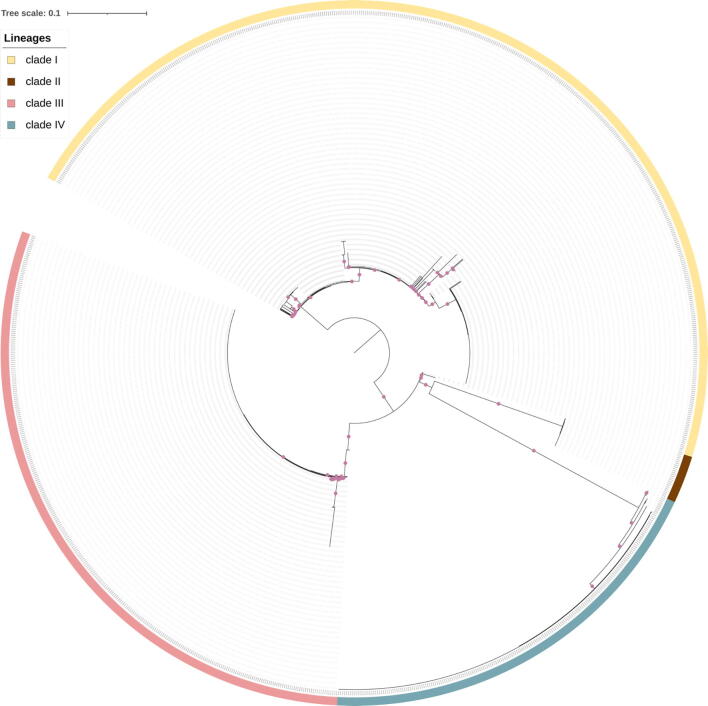
Neighbour-joining tree based on mitochondrial genomes showing relationships among 1,070 isolates. The color strips around the periphery indicate the clade affiliation of the strains based on their nuclear genome SNPs. Nodes with local support value over 0.75 were marked with a purple dot. (For interpretation of the references to color in this figure legend, the reader is referred to the web version of this article.)

Based on mitogenome SNPs, Misas et al. [69] clustered 130 *C. auris* strains into four clades, consistent with the clade affiliations based on nuclear SNPs. In their analyses, Clade III and Clade IV samples contained no SNP in their mitogenomes [69]. Furthermore, only one Clade II strain was included in their study, thus precluding the assessment of mitogenome variation within Clade II. Here, we included all *C. auris* strains with whole-genome sequences deposited in NCBI up to May 2022 in our analyses to determine whether including extra strains will reveal a cytonuclear phylogenetic pattern and a mitogenome variation pattern different from those reported by Misas et al [69]. Our phylogenetic analysis revealed that the mitochondrial and nuclear genomes showed a congruent clustering pattern at the clade level (Fig. 1), largely consistent with that observed in the previous study. Overall, this result suggests that at the clade level, there was no evidence for cytonuclear genome recombination among the four clades. However, there were several notable features. First, when using the Clade I strain B8441 mitogenome as the reference, several Clade IV strains had ambiguous nucleotide calls at several SNP loci, causing these strains having different branch lengths compared to other Clade IV strains on the phylogenetic tree. Second, all 24 Clade II strains analyzed here had the same mitogenome SNP pattern. Third, there were mitogenome SNPs within Clade III.

### 3.3 PI and LE analyses

The PI and LE analyses were conducted for each of the five datasets described in the Materials and Methods section. Below are brief summaries of the test results.

**Total sample:** For the total sample of 1,285 strains, 3.56% nuclear SNP pairs showed evidence of PI. Among these PI SNP pairs, 8.27% did not deviate significantly from random association. While the percentage of mitogenome SNP pairs being PI (3.29%) was similar to that of the nuclear genome, a much higher percentage of those mitochondrial SNP pairs (38.76%) were in LE than those nuclear SNP pairs (8.27%; Table 3). Together, the results suggest that in the total population of *C. auris*, both the nuclear and mitochondrial genomes showed low but unambiguous signatures of recombination with the mitogenome showed more frequent recombination than the nuclear genome.

**Table 3:** Nuclear and mitochondrial genome SNPs and signatures of recombination in the total population of *Candida auris*.

	Nuclear genome	Mitochondrial genome
Reference genome size (×10^6 bp)	12.37	0.028212
Sample size	1,285	1,285
Number of SNP sites	232,179	89
Total number of analyzed SNP pairs	26,953,427,931	3,916
SNP pairs in PI (% of total pairs)	959,299,294 (3.56%)	129 (3.29%)
SNP pairs in PI that fail to reject LE (% of total pairs that are in PI)	79,294,977 (8.27%)	50 (38.76%)

**Clade-specific nuclear genome analyses:** For the clade-specific nuclear SNPs, the percentages of SNP pairs being PI were 0.83%, 0.33%, 0.097%, and 1.36% for Clades I, II, III, and IV respectively (Table 4). These percentages were all lower than that in the total population shown in Table 3. Among the nuclear SNP pairs being PI, the percentages in LE were 77.95% (Clade I), 100% (Clade II), 88.10% (Clade III), and 81.11% (Clade IV) (Table 4), all higher than in the total population. Overall, the results are consistent with limited but unambiguous signatures of recombination within each of the four clades.

**Table 4:** Nuclear genome SNPs and signatures of recombination within each of the four clades of *C. auris*.

	Clade I	Clade II	Clade III	Clade IV
Reference nuclear genome size (×10^6 bp)	12.37	12.25	12.74	12.43
Sample size	537	24	514	210
Number of SNP sites	4,775	3,456	3,038	1,427
Total number of analyzed SNP pairs	11,397,925	5,970,240	4,613,203	1,017,451
SNP pairs in PI (% of total pairs)	94,327 (0.83%)	19,836 (0.33%)	4,496 (0.097%)	13,853 (1.36%)
SNP pairs in PI that fail to reject LE (% of total pairs that are in PI)	73,529 (77.95%)	19,836 (100%)	3,961 (88.10%)	11,237 (81.11%)

**Clade-specific mitogenome analyses:** We conducted similar tests to those described above for the mitogenome SNPs within the four individual clades. However, as described above, no unambiguous mitogenome variant was detected among strains within either Clade II or Clade IV. Thus, the PI and LE tests were conducted for only Clade I and Clade III samples. Among the 46 mitogenome SNP sites within Clade I, 0.68% (7/1,035) of all SNP pairs were in PI and two of the seven incompatible pairs were in LE (Table 5). However, no SNP pair was in PI among the 19 mitochondrial SNPs within Clade III (Table 5). Together, the results suggest evidence for mitogenome recombination among strains within Clade I but absent in other clades.

**Table 5:** Mitochondrial genome SNPs and signatures of recombination within Clades I and III. No SNP was found in the mitochondrial genomes of Clades II and IV.

	Clade I	Clade III
Reference mitochondrial genome size (×10^6 bp)	0.028212	0.028214
Sample size	537	514
Number of SNP sites	46	19
Total number of analyzed SNP pairs	1,035	171
SNP pairs in PI (% of total pairs)	7 (0.68%)	0
SNP pairs in PI that fail to reject linkage equilibrium (% of total pairs that are in PI)	2 (28.57%)	NA

**Clade-specific nuclear-mitochondrial SNP comparisons:** We further compared the nuclear SNPs with mitochondrial SNPs between each other within each clade. Here, three population samples were analyzed: the total sample, the Clade I sample, and the Clade III sample. Due to the lack of unambiguous mitochondrial SNPs within Clades II and IV, these two clades were not individually analyzed. For Clade I, we found that 1.0% of cytonuclear SNP pairs were in PI, among which 61.16% were in LE (Table 6). For Clade III, the percentage of cytonuclear SNP pairs in PI was 0.58%, among which 28.27% were in LE (Table 6). However, when the 232,179 nuclear SNPs were compared with all the 89 mitochondrial genome SNPs in the total sample, a much higher percentage (4.11%) of cytonuclear SNP pairs was in PI, and with 38.16% of them in LE (Table 6). Together, the results indicate that even though the mitochondrial and nuclear genomes showed overall congruent phylogeny at the clade level, at the individual SNP pair level, there was evidence of cytonuclear recombination/hybridization in both the total sample as well as in Clades I and III.

**Table 6:** Signatures of recombination based on mitochondrial and nuclear SNP comparisons.

	Clade I	Clade III	All four clades
Reference genome size (nuclear; mitochondrial, ×10^6 bp)	12.37; 0.028212	12.74; 0.028214	12.37; 0.028212
Sample size	537	514	1,285
Nuclear SNPs; mitochondrial SNPs	4,775; 46	3,038; 19	232,179; 89
Total number of analyzed SNP pairs	219,650	57,722	20,663,931
SNP pairs in PI (% of total pairs)	2,186 (1.0%)	336 (0.58%)	848,648 (4.11%)
SNP pairs in PI that fail to reject LE (% of total pairs that are in PI)	1,337 (61.16%)	95 (28.27%)	323,863 (38.16%)

### 3.4 Potential recombinogenic loci

**Fig. 2:**
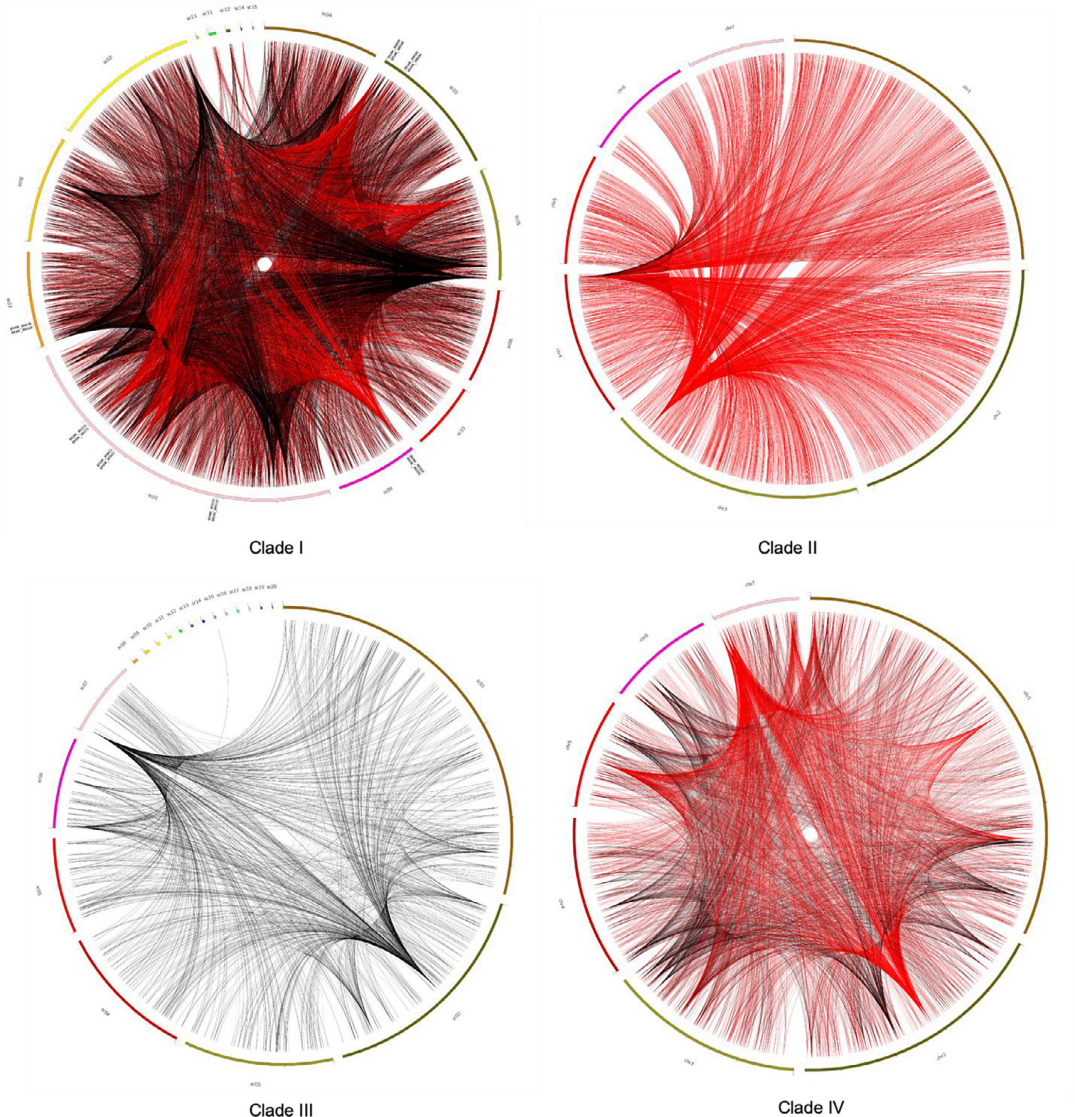
Circular plot showing genomic locations and links of SNP pairs in PI for each of the four clades. Highlighted in red are the SNP pairs with PI involving putative recombinogenic SNPs. (For interpretation of the references to color in this figure legend, the reader is referred to the web version of this article.)

Our analyses above revealed relatively low percentages of SNP pairs being PI in the nuclear genome, in the mitochondrial genome, and between the nuclear-mitochondrial genomes in individual clades. Here we are interested in identifying the top SNPs showing PI. In this analysis, we used a threshold of 20% as the cut-off to identify SNPs as high-frequency recombinant SNPs (i.e., the recombinogenic SNPs). Specifically, if a SNP was in PI with over 20% of the total SNPs in the population, the SNP is considered as putative recombinogenic SNP. Our search found that 20, 8, 0, and 23 SNP sites were recombinogenic in Clades I, II, III, and IV respectively. Overall, Clades II and III, the two *MTLα* clades, showed fewer recombinogenic SNPs than the two *MTLa* Clades I and IV (Fig. 2). Annotation of these SNP sites indicated that all the recombinogenic SNP sites are present in intergenic regions except one located in an intron. Comparison of the recombinogenic SNP sites among Clades I, II and IV identified two regions shared by all three clades. These are the intergenic regions between B9J08_000508 and B9J08_000509; and between B9J08_002254 and B9J08_002255. The GO annotations of the SNP flanking genes were extracted from the *Candida* genome database [70] and presented in Table S3.

### 3.5 Clade-shared SNP region

Among the 232,179 nuclear genome SNPs, 13 were polymorphic within each of the four clades. These 13 SNPs were located in three genomic regions and all three regions were intergenic (Table 7). Two of the three clade-shared SNP regions were the same as the “recombinogenic” regions identified above. The upstream gene of one region (region #2 in Table 7) has no known function while its downstream gene as well as both the upstream and downstream genes for the two remaining regions (regions #1 and #3) contain domains of genes known to be involved in multiple biological processes (Table 7). Two of the three regions (region 1 and region 2) overlapped with the recombinogenic regions identified above. However, excluding SNPs in these three regions from PI and LE testing had a minor impact on the total number and frequency of SNPs showing evidence for recombination. FGT of the 13 SNPs for each of the five datasets revealed relatively high levels of PI within Clade I (67/78 = 85.90%), Clade II (2/78 = 2.56%), Clade III (48/78 = 61.54%), Clade IV (33/78 = 42.31%), and the total sample (67/78 = 85.90%) and a very low level in Clade II (2/78 = 2.56%). We found that 12 of 67 PI SNP pairs in Clade I were in LE. For Clades II and III and the total samples, all PI SNP pairs were in linkage disequilibrium. In Clade IV, 15.15% of the PI SNP pairs were in LE.

**Table 7:** Details of the 13 clade-shared SNPs and the genes located around them.

	SNP loci	Ref	Alt	Upstream gene	Annotation	Downstream gene	Annotation
Clade-shared SNP region 1 (PEKT02000002.1)	9130	G	A	B9J08_000509: 12915-10831	Has domain(s) with predicted hydrolase activity	B9J08_000508: 8454-6094	Ortholog(s) have role in negative regulation of *TORC1* signaling and cytoplasm localization
	9137	C	T				
	9157	T	A				
	9175	G	A				
Clade-shared SNP region 2 (PEKT02000006.1)	48,506	T	A	B9J08_002255: 49410-49006	Protein of unknown function	B9J08_002254: 47801-47169	Has domain(s) with predicted FMN binding, oxidoreductase activity
	48,561	G	A				
	48,573	G	A				
	48,580	G	A				
	48,583	G	A				
	48,586	G	A				
Clade-shared SNP region 3 (PEKT02000007.1)	2,433,109	G	A	B9J08_003771: 2429975-2431693	Ortholog(s) have unfolded protein binding activity, role in protein folding and chaperonin-containing T-complex localization	B9J08_003772: 2436486-2436992	Has domain(s) with predicted DNA-binding transcription factor activity, RNA polymerase II-specific, zinc ion binding activity, role in regulation of transcription, DNA-templated and nucleus localization
	2,433,112	C	T				
	2,433,123	A	C				

### 3.6 Antifungal resistance related mutations

PI could be caused by parallel mutation or recombination. Parallel mutation is more likely to be selected and maintained in the population if they confer selective advantages. To investigate whether parallel mutations contribute to the observed PI, we selected genes previously identified or suspected as related to antifungal resistance and analyzed the associations among their SNPs. Specifically, the following genes were included in our analyses: *FUR, TAC1B, ERG3, ERG5, CDR1, PMS1, HOG1 ERG7, ERG11, STE6, YMC1, PDR1,* and *MSH2*. Here we hypothesize that if parallel mutations were responsible for the observed PI, we would see a significantly higher percentages of SNPs in these drug resistance related genes being PI than those in other parts of the genome. A total of 801 SNPs were identified among 1,285 strains in these 13 genes. Interestingly, among the 801 SNPs, 10,029 SNP pairs (out of 320,400 total pairs; 3.13%) showed PI. This rate is similar to that found between all SNPs in the nuclear genome (3.56%, Table 3). Of the 10,029 SNP pairs in PI, only 631 (6.29%) did not deviate significantly from random association, a percentage lower than that found for all SNPs in the nuclear genome (8.27%, Table 3). The results here suggest no evidence of higher frequency of parallel mutation at the antifungal resistance-associated genes in *C. auris*.

We further investigated whether similar rates of PI were also present among SNPs in these drug-resistance related genes for each of the four clades. After removing the fixed SNPs within each clade from the total SNPs identified above, 36, 23, 16, and 36 SNPs were identified in the 13 genes for Clades I, II, III, and IV respectively. Details about these mutations are listed in Supplementary Table S2. Only 3 pairs of antifungal related SNPs showed PI in Clade I, and with all three pairs in linkage disequilibrium. No evidence for PI between antifungal related SNPs was found for Clades II and III. For Clade IV, 75 of the 630 SNP pairs (11.90%) showed evidence for PI and with 71 of the 75 SNP pairs (94.67%) did not deviate significantly from LE. In addition, the exclusion of these SNPs from each of the clades had limited impact on the frequencies of SNP pairs that are in PI.

Together, our analyses indicate the possibility of antifungal pressure (and potentially other stresses) to select for parallel mutations across the clades to account for the observed PI, especially for Clade IV. However, such selection cannot explain most of the observed PI across the clades. In addition, none of the SNPs in the drug resistance-related genes, including those in Clade IV, belonged to the recombinogenic SNPs nor were they shared among all four clades.

## 4 Discussions

In this study, we analyzed the whole genome SNPs of 1,286 *C. auris* strains collected from across the world over the past 20+ years to investigate the potential signatures of recombination in this species. SNPs in both the nuclear and mitochondrial genomes were analyzed, both in the total sample as well as for each of the four clades where multiple strains have been sequenced. Our analyses revealed signatures of infrequent recombination in both the total sample as well as within each of the four individual clades. In addition, specific groups of SNPs, including those in genes involved in antifungal drug resistance as well as those that are shared among all four clades, were separately analyzed to help identify the potential contributors to the observed signatures of recombination. Different patterns of allelic associations were found among the sample types and between the nuclear and mitochondrial genomes. Below we discuss the main findings of our analyses and the major implications of our results.

### 4.1 Comparison between nuclear and mitochondrial genomes

The 1,285 genomes analyzed here represented all the strains of *C. auris* that have been sequenced and deposited in GenBank by researchers, up to May 2022. Multiple studies have analyzed variable numbers of strains, with the largest number of strains analyzed by Muñoz et al. [20] where 304 strains from many geographic regions were included. Most studies have focused on nuclear genomes. Analyses of nuclear genomes in those studies revealed that the global population of *C. auris* could be grouped into five distinct clades, with Clades I to IV represented by multiple strains in each while Clade V was represented by only one whole-genome sequenced strain (so far). However, one previous study analyzed mitogenome variations. Based on mitogenome SNPs of 130 *C. auris* strains, Misas et al. [69] showed that the mitogenome and nuclear genome SNPs clustered the strains into four similar clades. However, their analyses included only one Clade II strain and their results based on 10 Clade III strains from South Africa revealed no mitogenome sequence variation within Clade III. Our analyses here significantly expanded the sample sizes of all four clades with a total of 1,285 strains. While a similar pattern of sequence divergence within *C. auris* into five clades for both the nuclear genome and the mitochondrial genome was observed as previously reported (20,70), our analyses also revealed several notable features. Specifically, first, despite having more than twice as many strains as the earlier study (210 vs 86), we found no unambiguous SNP within the mitogenome of Clade IV, similar to that found by Misas et al. [69]. Second, the inclusion of 23 additional Clade II strains (versis one strain in the Misas et al. study) revealed no mitogenome SNP within Clade II. Third, the inclusion of 504 additional strains from more diverse geographic sources in Clade III (i.e., 514 in this study vs 10 in the Misas et al study) revealed abundant mitogenome SNPs within Clade III. Together, these analyses revealed that the amounts of sequence variations between the nuclear and mitochondrial genomes differed at both the whole species as well as within individual clades. At the whole species level, the SNP frequency in the nuclear genome was 1.876% (232,179/12.37×10^6^), about six times of that of the mitochondrial genome (0.315%; 89/28212).

The lower observed genetic variation in the mitochondrial genome than in the nuclear genome has been reported in several other fungal species, including the human pathogen *Cryptococcus gattii* species complex and the ectomycorrhizal mushroom *Tricholoma matsutake* species complex [71], [72], [73]. However within individual clades, while two clades (Clades II and IV) showed limited to no mitochondrial SNPs (consistent with the overall pattern within the species), the remaining two clades (Clades I and III) showed greater mitochondrial SNP frequencies than their respective nuclear genomes. At present, the mechanisms for the different amounts of sequence diversity between the two genomes among the clades are unknown. The small sample size and limited ecological niches (mostly from ear discharges) might have contributed to no mitochondrial sequence variation in Clade II. However, this explanation cannot hold for Clade IV where 210 strains from four continents and a variety of human body sites were examined, similar to those of Clades I and III strains in this study (Table S1). Geographically, strains of Clades II and IV are predominantly found in East Asia and the Americas respectively while Clades I and III are predominantly from South Asia and Africa respectively. It is possible that the higher temperature and other potential environmental factors in South Asia and Africa may have contributed to the higher mutation rates in mitochondrial genomes than in nuclear genomes in Clades I and III. The mechanisms for the observed divergent mitochondrial vs nuclear genetic variations among the four clades within *C. auris* remain to be elucidated.

### 4.2 Evidence of recombination

At both the species and individual clade levels, though the frequencies were generally low, evidence for PI was observed in both the nuclear and the mitochondrial genomes, with a significant proportion of those PI SNP pairs also in linkage equilibrium. However, the frequencies of PI SNP pairs differed among the samples. Overall, the frequency of nuclear PI SNP pairs at the species level was from twice to over 30 times of those within individual clades (Table 3, Table 4). A similar pattern was also observed for the mitochondrial genome SNPs. Together, these results suggested that there was more frequent recombination before the divergence of the clades than after individual clades were established. Specifically, though signatures of recombination were also detected within each of the four clades after their respective divergence, clonal reproduction and expansion seemed more dominant in natural populations of *C. auris* after the divergence of clades than before their divergence. Our observed pattern is largely consistent with the expectations of each clade having only one mating type and therefore less likely to mate and recombine among strains of the same clade.

Relative to the frequent reports of recombination in the nuclear genomes of fungal populations, reports of mitochondrial genome recombination are still rare. However, the list of fungal species and populations showing evidence of mitogenome recombination is growing. For example, since 1998, mitochondrial DNA recombination has been reported for the honey mushroom *Armillaria gallica* [74]*,* the commercial button mushroom *Agaricus bisporus* [75], the wild ectomycorrhizal mushroom *Russula virescens* species complex [76], and the opportunistic human fungal pathogen *Cryptococcus gattii* species complex [71]. In the commercial mushroom *A. bisporus*, the observed frequency of mitochondrial loci with PI was correlated with the life cycles of two varieties within the species, with the outcrossing heterothallic population showing more evidence of mitochondrial genome recombination than the secondarily homothallic populations [75].

Because the ancestral population of *C. auris* contained strains of both mating types, evidence for recombination in the total sample was expected. The higher rate of SNP pairs that showed evidence for PI than those within individual clades is consistent with sexual recombination in the ancient population of this species. The absence of incongruent relationships among clades between nuclear and mitochondrial genome phylogenies is consistent with the absence of mating and recombination among the four clades after their divergence from each other. However, the observed PIs among SNP pairs within individual clades after their divergence are puzzling. Specifically, each clade is known to contain strains of only one mating type, *MTLa* for Clades I and IV, and *MTLα* for Clades II and III. In addition, we found limited evidence of parallel mutations in the genes that are most likely under parallel selective pressure, the antifungal drug resistance-related genes. While we cannot completely exclude the possibility that convergent mutations might have contributed to some of the observed PIs, our analyses revealed that even if they existed, such an effect would likely be minimal. However, evidence for recombination have been found in natural fungal populations known to contain only a single mating type. For example, same-sex mating has been reported in the human fungal pathogen *Cryptococcus neoformans* species complex and such mating can generate genetic recombinants, similar to what have been reported for opposite-sex mating and to natural populations containing strains of both mating types [77]. It is possible that low-frequency same-sex mating could have similarly happened to the individual *C. auris* clades to generate the observed PIs and linkage equilibrium. Alternatively, low frequency strains of the alternative mating type may exist within each of the four clades in nature and mating between strains of opposite mating types could have generated the observed signatures of recombination. Indeed, these two possibilities are not mutually exclusive, and both could have contributed to the observed signatures of recombination. Broader and more intensive sampling as well as experimental investigations of genetic crosses are needed in order to test these two possibilities.

### 4.3 Genes adjacent to clade-shared SNPs

Our analyses revealed three clade-shared SNP regions, with SNPs in two of these regions showing high frequency of PI with other SNPs in the genome. Interestingly, all three clade-shared SNP regions are in intergenic regions between genes coding for hydrolases, oxidoreductases, and transcription factors with potential impacts on cell growth and lifespan (Table 7). For example, the ortholog of B9J08_000508, the downstream gene of clade-shared SNP region 1, is known to regulate the target of rapamycin complex 1 (*TORC1*) signaling. *TORC1* is a multiprotein signaling complex functions as the organizer that incorporate internal and external cues to regulate cell growth and cell cycle progression [78]. A recent study demonstrated that *TORC1* signaling plays an important role in controlling NaCl resistance through *Sir2* in *Saccharomyces cerevisiae* [79]. The clade-shared SNPs within the upstream region of *TORC1* gene may be involved in regulating the expression levels of *TORC1*.

Interestingly, the downstream gene of clade-shared SNP region 2, B9J08_002254, codes for a protein containing a putative FMN-binding domain which is known to be most frequently found in bacteria. It has been hypothesized that proteins containing such a domain in fungi may have been horizontally transferred from bacterial to fungal genomes [80]. Indeed, multiple independent transfers of such genes and the associated upstream sequences from bacteria to strains of *C. auris* in different clades could have contributed to the observed distributions of clade-shared polymorphisms and PIs. Our BLAST searches revealed that based on the amino acid sequence, the closest match to B9J08_002254 was in the bacterial genus *Achromobacter,* with a 99% query coverage and an E value of 1e-54.

The two genes located upstream and downstream of the clade-shared SNP region #3 were B9J08_003771 and B9J08_003772. Gene B9J08_003771 has a predicted unfolded protein-binding activity, while B9J08_003772 has a predicted DNA-binding transcription factor activity, zinc ion binding activity, and transcriptional regulation activity. The unfolded protein response is known to help human fungal pathogens survive in the host through balancing the load of proteins entering the endoplasmic reticulum and the protein-folding capacity of the organelle [81]. For example, in *C. albicans*, the zinc finger protein *CZF1* is one of the DNA-binding proteins of the Cys6Zn2 class of transcriptional regulators with a multitude of functions such as biofilm induction, hyphal growth regulation, white-opaque switch, and yeast cell adherence [82], [83], [84], [85], [86]. While functionally likely important, how the polymorphisms in the intergenic regions of these two genes contribute to strain and population fitness remains to be investigated.

### 4.4 Conclusions and perspectives

This study identified limited but unambiguous evidence of recombination in both the total sample and within individual clades. In addition, evidence of recombination was found in both the nuclear and mitochondrial genomes, as well as between the nuclear and mitochondrial genomes. Overall, signatures of recombination were more prominent in the total sample than within individual clades, consistent with greater frequencies of recombination before the divergence of the four clades than after their divergence. At present, while several possibilities were suggested, the mechanism(s) for the observed recombination is not known. Nevertheless, the signatures of recombination identified here suggested a number of avenues from which further investigations could be conducted, including more extensive sampling for alternative mating types within each clade, laboratory attempts of both same-sex and opposite-sex mating, and identifying the adaptive significance of clade-shared SNPs. Such investigations should allow us to better understand the genetic architecture of virulence and drug resistance evolution within and among the divergent clades of this pathogen in natural and clinical environments.

## Funding

This research was supported by grants from the Natural Sciences and Engineering Research Council of Canada (Grant No. CRDPJ 474638-14) and the Faculty of Science’s Global Science Initiative of McMaster University. Y.W. is supported by a MacData Fellowship.

## Conflicts

The authors declare no conflict of interest.
